# Disseminated Histoplasmosis in HIV-Infected Patients: A Description of 34 Years of Clinical and Therapeutic Practice

**DOI:** 10.3390/jof6030164

**Published:** 2020-09-07

**Authors:** Mathieu Nacher, Audrey Valdes, Antoine Adenis, Romain Blaizot, Philippe Abboud, Magalie Demar, Félix Djossou, Loïc Epelboin, Caroline Misslin, Balthazar Ntab, Kinan Drak Alsibai, Pierre Couppié

**Affiliations:** 1 CIC INSERM 1424, Centre Hospitalier Andree Rosemon Cayenne, 97300 Cayenne, French Guiana, France; antoine.adenis@ch-cayenne.fr; 2 Département Formation Recherche Santé, Université de Guyane, Cayenne, 97300 Cayenne, French Guiana, France; romain.blaizot@ch-cayenne.fr (R.B.); pierre.couppie@ch-cayenne.fr (P.C.); 3 Equipe Opérationnelle d’Hygiène Hospitalière, Centre Hospitalier Andree Rosemon Cayenne, 97300 Cayenne, French Guiana, France; audrey.valdes@ch-cayenne.fr; 4 Department of Dermatology, Centre Hospitalier Andree Rosemon Cayenne, 97300 Cayenne, French Guiana, France; 5 Service des Maladies Infectieuses et Tropicales, Centre Hospitalier Andree Rosemon Cayenne, 97300 Cayenne, French Guiana, France; philippe.abboud@ch-cayenne.fr (P.A.); felix.djossou@ch-cayenne.fr (F.D.); loic.epelboin@ch-cayenne.fr (L.E.); 6 Laboratory, Centre Hospitalier Andree Rosemon Cayenne, 97300 Cayenne, French Guiana, France; magalie.demar@ch-cayenne.fr; 7 Unité Mixte de Recherche Tropical Biome and Immunopathology, Université de Guyane, 97300 Cayenne, French Guiana, France; 8 Service de Médecine, Centre Hospitalier de l’Ouest Guyanais, 97320 Saint Laurent du Maroni, French Guiana, France; c.misslin@ch-ouestguyane.fr; 9 Département d’Information Médicale, Centre Hospitalier de l’Ouest Guyanais, 97320 Saint Laurent du Maroni, French Guiana, France; b.ntab@ch-ouestguyane.fr; 10 Department of Pathology, Centre Hospitalier Andrée Rosemon, 97300 Cayenne, French Guiana, France; Kdrak.alsibai@doctor.com

**Keywords:** disseminated histoplasmosis, HIV, treatment, case fatality, liposomal amphotericin B, French Guiana

## Abstract

Disseminated histoplasmosis is the main AIDS-defining infection of French Guiana. We aim to describe our therapeutic experience for 349 patients with disseminated histoplasmosis between 1 January 1981 and 10 January 2014 in French Guiana. Survival, delays for treatment initiation, duration of induction therapy, and associated initial treatments are described. The death rate was 14.9 per 100 person-years, with an early drop in survival. Among those who died, >1/3 died within a year of HIV diagnosis, and ¾ of all patients with histoplasmosis had been diagnosed for HIV within a year. As induction treatment, 29% received liposomal amphotericin B, 12.9% received deoxycholate amphotericin B, 54% received itraconazole alone, and 21.8% received liposomal amphotericin B and itraconazole. The median delay between symptoms-onset and hospitalization was 19.5 days (IQR = 5–105). Liposomal amphotericin B or itraconazole was initiated shortly after admission. Treatment initiation was often presumptive for liposomal amphotericin B (27%) and itraconazole (20%). The median duration of liposomal amphotericin B treatment was 7 days (IQR = 5–11 days). The present study shows that ¾ of the patients were profoundly immunocompromised and had been diagnosed for HIV within the past year. Antifungal treatment was often initiated presumptively on admission. Over time there was a significant gradual decline in early death.

## 1. Introduction

The HIV epidemic has grown since the early 1980s in French Guiana, a French overseas territory between Brazil and Suriname. One of the greatest particularities of patients with advanced HIV in French Guiana is the high incidence of disseminated histoplasmosis, which is the most frequent AIDS-defining infection and cause of death among HIV-infected patients [1,2]. Awareness of this epidemiological fact has grown since the 1980s, starting with the dermatologists, then spreading to all physicians, when the implementation of fungal culture increasingly allowed the identification of the fungal pathogen [3,4]. Hence, within three decades, case fatality at one month dropped from 40% of cases to less than 4% [2]. In Latin America, the frequent lack of awareness about histoplasmosis and the lack of availability of diagnostic methods translate into thousands of annual deaths among persons with advanced HIV [5]. For moderately severe to severe disseminated histoplasmosis, the updated 2007 guidelines of the Infectious Diseases Society of America [6,7] recommend induction with liposomal amphotericin B for 1–2 weeks, followed by oral itraconazole for a total of at least 12 months. For mild-to-moderate histoplasmosis, itraconazole is recommended for 12 months. In 2020, to fight the high burden of histoplasmosis-related deaths in persons with advanced HIV, PAHO also developed guidelines for the diagnosis and treatment of disseminated histoplasmosis [8].

Between crisp written clinical guidelines and real life, there is often a gap. In practice, at the bedside, physicians are often guided by clinical and empirical approaches that may diverge from recommendations. For countries where rapid diagnostic methods are absent, reliance on slow fungal growth to identify *Histoplasma* often leads to great delays in diagnosis, hence requiring presumptive treatment to avoid the detrimental consequences of untreated histoplasmosis. In this context, we aim to describe our therapeutic experience in French Guiana, notably, the delays for treatment initiation, the duration of induction therapy, and the associated initial treatments. 

## 2. Methods

### 2.1. Study Design

A retrospective, observational, multicenter study was performed on patients included between 1 January 1981 and 1 October 2014.

### 2.2. Study Population

The study population consisted of coinfections with HIV and histoplasmosis that were enrolled in the Histoplasmosis and HIV database of French Guiana. The inclusion criteria were confirmed HIV infection, first proven episode of histoplasmosis (direct mycological examination, culture mycological, or histological examination (excluding PCR) performed on different samples (plasma, bone marrow, digestive biopsies, skin biopsies, bronchoalveolar lavage) following the EORTC/MSG criteria [9], and age >18 years. Histoplasmosis that was not proven (successful empirical antifungal therapy) or diagnoses based solely on the positivity of PCR or recurrent histoplasmosis was not considered.

### 2.3. Study Conduct

The HIV–Histoplasmosis database was created in 1992. Incident cases of HIV-associated histoplasmosis in infected patients in the three hospitals of French Guiana were included. Epidemiological, clinical, paraclinical, immunovirological, and therapeutic data were collected on a standardized paper form until October 2014. Hospitalized incident episodes of HIV-associated histoplasmosis (known to be HIV-positive or concomitantly discovered) were included. The recorded variables were sociodemographic data (sex, age, place of birth), clinical data (symptoms on admission, clinical entrance examination, immunovirological assessment, standard biological examinations, medical imaging, mycology, pathology, treatment received, duration, dosage, route of administration), and survival data, were collected which during the study period. The cohort is not funded and, throughout time, has relied on physicians accepting the task of collecting medical records and searching for laboratory results, ascertaining the vital status of patients. The reason why cases after 2014 are not included is the lack of dedicated staff.

### 2.4. Statistical Analysis

The statistical analysis was performed with STATA^©^ (College Station, TX, USA). Quantitative variables were described using medians and interquartile ranges; they were compared between groups using rank-sum nonparametric tests.

Survival analysis was also performed with different failure events (death, treatment onset, discharge) and different entry points (beginning of symptoms, date of hospitalization).

Kaplan–Meier curves were plotted, incidence rates were computed, and simple Cox proportional hazards were calculated. 

### 2.5. Ethical and Regulatory Aspects

The 1992 Histoplasmosis and HIV anonymized database was approved by the Comité Consultatif pour le Traitement de l’Information pour la Recherche en Santé (CCTIRS; N° 10.175bis, 10 June 2010), the French National Institute of Health and Medical Research institutional review board (CEEI INSERM; IRB0000388, FWA00005831 18 May 2010), and the Commission Nationale Informatique et Libertés (CNIL; N° JZU0048856X, 16 July 2010).

## 3. Results

### 3.1. General Results and Presentation

Overall, there were 349 cases of HIV-associated disseminated histoplasmosis. The mean age was 40 (±9.7) years. The sex ratio was 1.9 males per female. The median CD4 count was 31 (IQR = 12–70) CD4 lymphocytes per mm3. The most frequent presentations were alteration of the WHO general performance status, fever, digestive tract symptomatology, respiratory symptomatology, and lymphadenopathies (Table 1). 

### 3.2. Concomitant AIDS-Defining Events 

Among patients, 137 (39%) had a concomitant opportunistic infection. Overall, among the 349 patients with disseminated histoplasmosis, 26 had esophageal candidiasis, 20 had chronic herpes, 18 had tuberculosis, 18 had cerebral toxoplasmosis, 15 had bacteriemia, 8 had atypical mycobacterial infection, 8 had salmonella infection, 7 had pneumocystosis, 3 had chronic diarrhea with cryptosporidia, 2 had cryptococcosis, 2 had recurrent bacterial pneumopathy, 1 had cytomegalovirus infection, and 1 had aspergillosis. In addition, 1 had Kaposi syndrome, 2 had lymphoma, and 6 had HIV nephropathy. 

### 3.3. Hospitalization Duration

**Figure 1:**
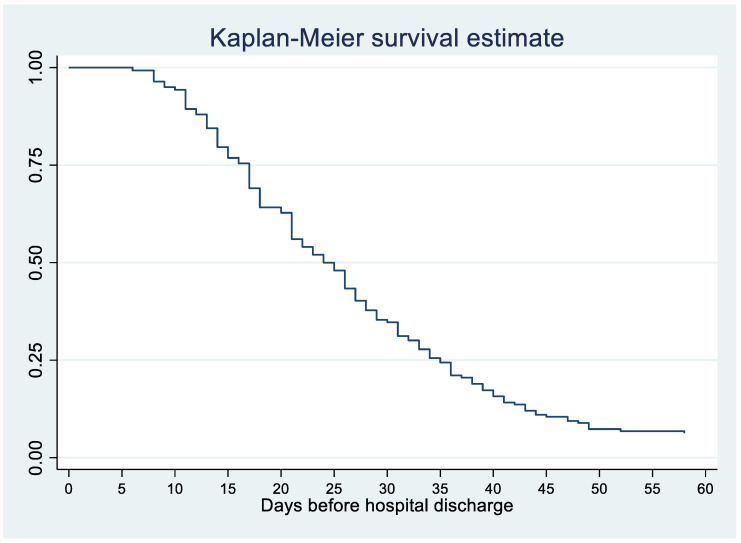
HIV-associated disseminated histoplasmosis: Days in hospital before discharge or death. 1981–2014, French Guiana.

Figure 1 shows that a quarter of patients with disseminated histoplasmosis stayed <16 days, half stayed less than 25 days, ¾ stayed between 25 and 35 days, and ¼ stayed over 35 days.

### 3.4. Deaths 

**Figure 2:**
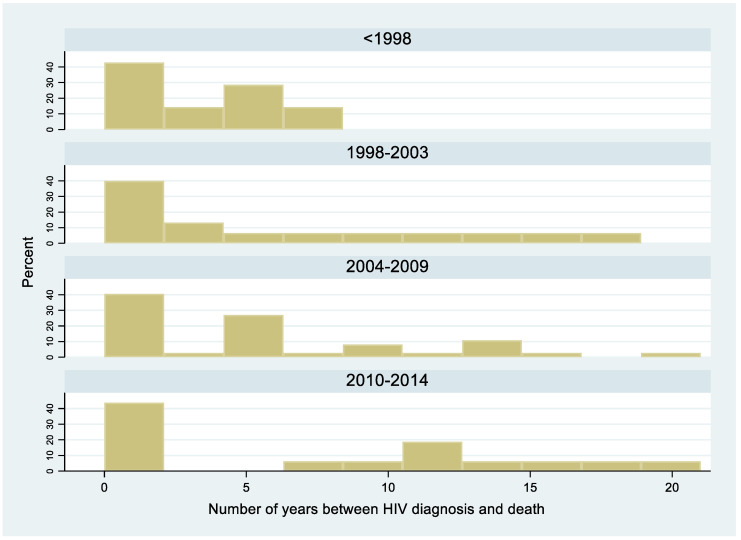
HIV-associated disseminated histoplasmosis: number of years between HIV diagnosis and death of any cause. 1981–2014, French Guiana.

**Figure 3:**
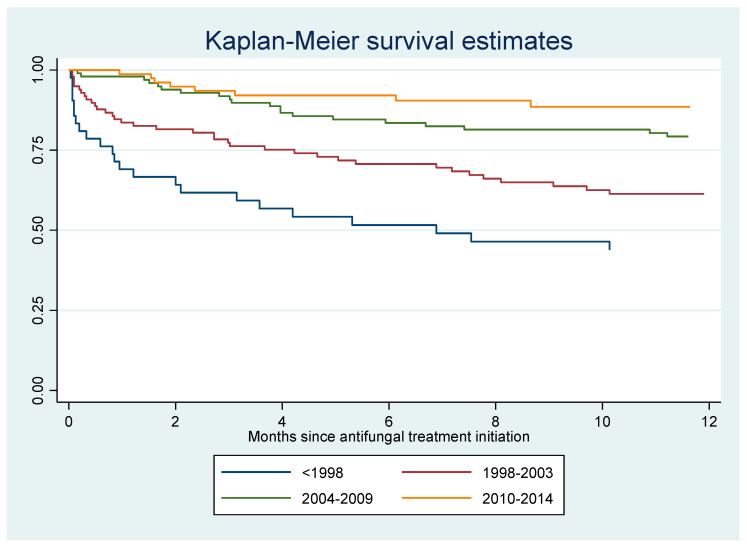
Incidence of death within 12 months after antifungal treatment initiation for HIV-associated disseminated histoplasmosis. 1981–2014, French Guiana.

Among the 349 cases, there were 144 deaths, among which 50 (14%) occurred within one month of antifungal therapy initiation. There was a gradual decline in early death (*p* < 0.0001, chi-square for trend): before 1998, 18/47 (38%) patients died within 1 month of antifungal treatment; between 1999 and 2003, 17/100 (17%) died; between 2004 and 2009, 10/111 (9%) died; between 2010 and 2014, 5/91 (5.5%) died. The incidence rate of death among HIV-infected patients with disseminated histoplasmosis was 14.9 per 100 person-years. Hence among deaths, without presuming the cause of death, a quarter of hospitalized patients died within a month, half within 102 days, nearly ¾ within a year of hospitalization for histoplasmosis. Figure 2 shows that over a third of patients died within a year of HIV diagnosis. The Kaplan–Meier curve for death for the first 12 months after antifungal treatment initiation shows an early drop corresponding to early deaths, followed by a gradual flattening (Figure 3). After 7 months, 25 percent of patients had died. The presence of a concomitant opportunistic infection was not associated with any significant differences in mortality. The presence of digestive signs and symptoms was associated with a decreased hazard of dying (HR = 0.7, 95% CI = 0.4–0.9, *p* = 0.01). Overall, the median duration between the onset of disseminated histoplasmosis and treatment induction was longer among those who died (124 days; IQR = 35–257) than among those who did not (31 days; IQR = 18–201, *p* < 0.0001).

### 3.5. Induction Treatments

Among the induction treatments of these 349 patients with disseminated histoplasmosis, 102 received liposomal amphotericin B, 45 received deoxycholate amphotericin B, 6 received fluconazole (4 IV, 2 oral), 269 received itraconazole (76 with liposomal amphotericin B, 3 with deoxycholate amphotericin B), 10 received rifampin (8 were on itraconazole), and 8 received rifabutin (6 were on itraconazole) for concomitant tuberculosis. There was a nonsignificant trend for lower CD4 counts in those receiving amphotericin (liposomal, deoxycholate) than those who did not: 29 (IQR = 10–65) vs. 33 (IQR = 15–74), respectively; *p* = 0.18. Overall, 6 patients received fluconazole, a drug with good diffusion within the central nervous system. Among these 6 patients, 5 had neurological signs, and one had associated cryptococcal meningitis.

### 3.6. Delay between Initial Symptoms and Liposomal Amphotericin B Initiation and between Hospitalization and Liposomal Amphotericin B Initiation 

**Figure 4:**
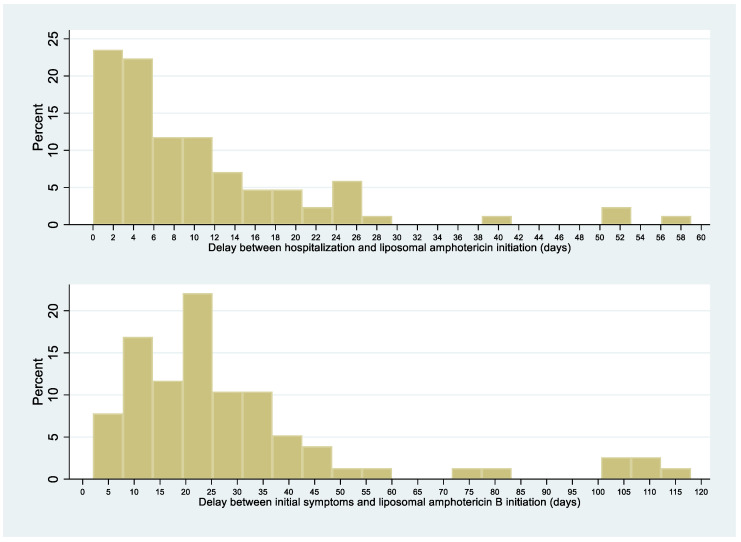
HIV-associated disseminated histoplasmosis: delay between initial symptoms, hospitalization, and liposomal amphotericin B initiation.

The median delay between symptoms-onset and hospitalization was 19.5 days (range, 5–105). There was no significant difference in this median delay between patients dying within 1 month of antifungal therapy or surviving until that date (21 (IQR = 4–100) vs. 19 (IQR = 5–107), respectively; *p* = 0.9). For half of the patients, liposomal amphotericin B was initiated within 7 days after admission; for 25% of patients, within 3 days after admission (Figure 4). Over 4 time periods (<1998, 1998–2003, 2004–2009, 2010–2014), the interval between initial symptoms and histoplasmosis treatment decreased over time (median regression, *p* < 0.001). The interval between initial symptoms and diagnosis also decreased significantly and linearly over time (median regression, *p* < 0.001).

### 3.7. Median Delay between Diagnosis and Liposomal Amphotericin B Initiation

For 101 patients with complete information, the median delay between diagnosis and liposomal amphotericin B initiation was zero-days; 28/101 of patients were treated with liposomal amphotericin B before diagnostic confirmation, and, for those patients, the median was 15 days before diagnostic confirmation.

### 3.8. Median Duration of Liposomal Amphotericin B Treatment 

**Figure 5:**
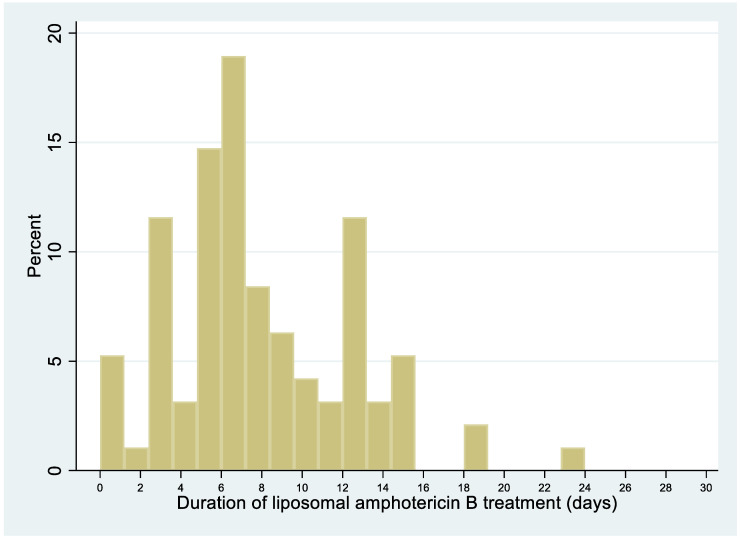
HIV-associated disseminated histoplasmosis: duration of liposomal amphotericin B treatment.

The median duration of liposomal amphotericin B treatment was 7 days (IQR = 5–11 days; Figure 5).

### 3.9. Delay between Onset of Disseminated Histoplasmosis Symptoms and Itraconazole Initiation

**Figure 6:**
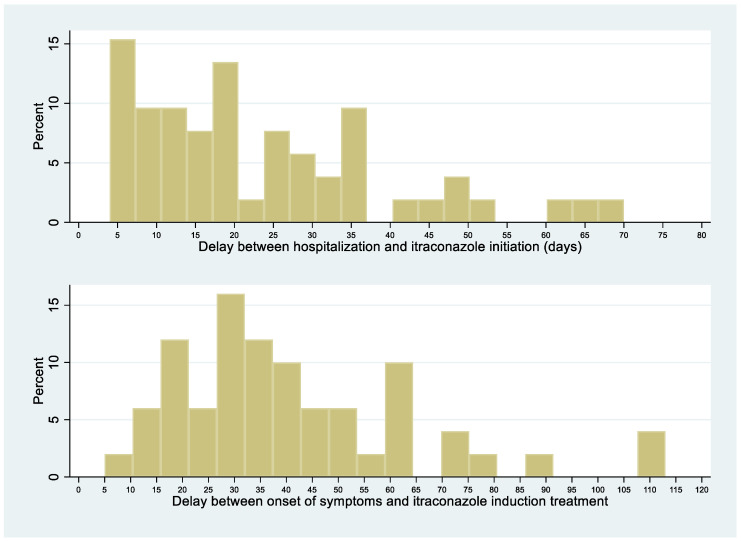
HIV-associated disseminated histoplasmosis: delay between initial symptoms, hospitalization, and itraconazole initiation.

Figure 6 shows the delay Between Onset of Disseminated Histoplasmosis Symptoms and Itraconazole Initiation. The median delay between the first symptoms of disseminated histoplasmosis and itraconazole initiation was 41 days. Among 61 patients for whom the information was available, 12 (20%) had received itraconazole before the diagnosis of disseminated histoplasmosis was ascertained, with a median of 11 days before fungal confirmation.

## 4. Discussion

The present study describes 34 years of clinical and therapeutic practice of disseminated histoplasmosis in French Guiana. Overall, 39% of patients with disseminated histoplasmosis had a concomitant opportunistic infection, mostly esophageal candidiasis, chronic herpes, tuberculosis, and cerebral toxoplasmosis. Half of the patients were hospitalized for over 25 days, and 50 patients died within one month of antifungal treatment initiation. Although the one-month threshold is usually used to attribute deaths to histoplasmosis, the Kaplan–Meier curve showed that the death rate initially increased rapidly, and then the curve flattened, with 25 percent of patients dying after 7 months. Among those who died, a quarter had died within a month, half within 102 days, and nearly ¾ within a year of hospitalization for histoplasmosis, representing an incidence rate for death of 14.8 per 100 person-years. Among those who died, over a third of patients had died within a year of HIV diagnosis, and ¾ of all patients with histoplasmosis had been diagnosed for HIV within a year, emphasizing the importance of late HIV diagnoses as the major contributor to disseminated histoplasmosis in French Guiana. It is, however, difficult to directly attribute death to histoplasmosis in profoundly immunocompromised patients, especially after the 1-month threshold. Nevertheless, even when other direct causes are involved, it is likely that such a disseminated infection constitutes an important contributive factor.

Among the induction treatments of these 349 patients with disseminated histoplasmosis, 29% received liposomal amphotericin B, 12.9% received deoxycholate amphotericin B, 54% received itraconazole alone, and 21.8% received both liposomal amphotericin B and itraconazole. The dual therapy approach is commonly utilized in French Guiana as the patient receives therapeutic amphotericin B during the necessary time for the achievement of appropriate itraconazole serum concentrations, and then the toxic and expensive amphotericin B is discontinued once the patient has improved. Rare patients with neurological manifestations received fluconazole, which is less effective against *Histoplasma* than itraconazole, but it has excellent penetration into the central nervous system. Five percent had concomitant tuberculosis treated with rifampin or rifabutin, and most of these patients were also receiving itraconazole, usually without performing drug concentration monitoring. There was no difference in case fatality between those with or without a concomitant opportunist infection.

The median delay between symptoms-onset and hospitalization was 19.5 days (range, 5–105). This delay seems both long for a progressive systemic infection but also short for what is a chronic problem. There was a clear link between the duration of the onset of symptoms and antifungal treatment induction, which raises the question as to why these people would only arrive in the hospital after weeks of symptomatic illness. The frequent ignorance of one’s HIV infection and delays in testing and accessing care are probable major contributors, as suggested by the above observation that ¾ of disseminated cases had been diagnosed with HIV for <1 year [10,11,12,13]. The subacute presentation suggested that dissemination of *H. capsulatum* was gradual and pernicious, and that awareness of HIV and awareness of histoplasmosis were required to obtain a rapid diagnosis. Over time, the delay between first symptoms and antifungal treatment initiation and between first symptoms and diagnosis of disseminated histoplasmosis gradually declined, presumably explaining, in part, the decline in case fatality in French Guiana. The symptoms presumably appear an undetermined amount of time after the fungal pathogen has started to spread. Hence, analogously to cryptococcosis, systematic screening could avoid treatment delays and control the infection before it becomes clinically evident [14]. For most patients, liposomal amphotericin B or itraconazole was initiated shortly after admission. Treatment initiation was often presumptive for liposomal amphotericin B (for 27%, a median of 15 days before diagnostic confirmation) and itraconazole (for 20%, a median of 11 days before diagnostic confirmation). However, in some patients, treatment initiation was much very late, perhaps because of nonspecific symptoms and diagnostic difficulties in earlier phases of dissemination. Indeed, it seems crucial to minimize delays in treating a disseminated infection, as shown by the significantly longer delays between symptoms-onset and antifungal treatment in patients who subsequently died than in those who did not. This requires awareness of the high probability of histoplasmosis in patients with advanced HIV disease in endemic areas [15,16]. In line with recommendations, the median duration of liposomal amphotericin B treatment was 7 days, with some variability (IQR = 5–11 days) [7]. Hence, some patients improved rapidly and were left on itraconazole alone; others did not improve and received longer regimens of liposomal amphotericin B.

The study limitations are linked to its retrospective nature and to the long time-span that encompassed changes in diagnostic methods (fungal culture in 1998) and therapeutics: until December 2003, deoxycholate amphotericin B was used on a central venous catheter (1 mg/kg/day) and, starting December 2003, liposomal amphotericin B (3 mg/kg/day) through a peripheral catheter replaced it [17]. However, this real-life experience over a time span of 34 years is also a strength, perhaps the largest cohort of HIV-associated disseminated histoplasmosis ever published. There are few data describing the management and outcome of disseminated histoplasmosis in South America and the Amazonian region. Perhaps the closest reports originate from Fortaleza in Brazil, who described risk factors for death [18].

In conclusion, the present study shows that ¾ of the patients were profoundly immunocompromised and had been diagnosed for HIV within the past year. Delays between symptoms-onset and antifungal treatment were associated with death; these delays gradually declined in French Guiana, presumably due to increased awareness and improved diagnosis. Antifungal treatment was often initiated presumptively on admission, and liposomal amphotericin B was widely used. Despite a significant 8-fold decline in early mortality, fatalities remained significant throughout the first year. HIV testing and antiretroviral treatment fail to reach all infected patients, and healthcare providers should strive to improve HIV testing and treatment in order to minimize the reservoir of undiagnosed and untreated HIV infections. Among immunocompromised persons, screening for histoplasmosis could also minimize the damage caused by the progressive dissemination of *H. capsulatum*.

## Figures and Tables

**Table 1:** Symptoms and signs for HIV-associated disseminated histoplasmosis. 1981–2014, French Guiana.

Variable	N	Proportion (%)
**Symptoms & signs**		
***Fever***	348	89
***Weigh loss***	158	83
***Digestive***	348	70
***Lymph node***	348	48
***Pulmonary***	348	47
***Neurological***	348	20
***Cutaneous***	348	6
***Oral***	348	5
***Occular***	348	0.28

Overall, 75% of patients had received their HIV diagnosis within a year.
